# TMAO and diabetes: from the gut feeling to the heart of the problem

**DOI:** 10.1038/s41387-025-00377-8

**Published:** 2025-05-20

**Authors:** Kinga Jaworska, Monika Kuś, Marcin Ufnal

**Affiliations:** Department of Experimental Physiology and Pathophysiology, Laboratory of the Centre for Preclinical Research, Medical University of Warsaw, Warsaw, Poland

**Keywords:** Diabetes, Preclinical research, Risk factors

## Abstract

Elevated plasma levels of trimethylamine N-oxide (TMAO)—a compound derived from diet and the gut microbiome—have been widely studied for their association with diabetes risk and their potential role in disease pathophysiology and complications. However, clinical studies, both prospective and retrospective, have yielded conflicting results. For example, elevated levels of TMAO are frequently linked to an increased risk of cardiovascular and renal complications in individuals with diabetes. However, the robustness and independence of these associations differ across study populations and are influenced by the degree of adjustment for confounding risk factors. Considering insulin’s regulatory effect on FMO3 activity in liver cells, TMAO may serve as a marker of hepatic insulin resistance, which could partially explain its association with diabetes risk. The role of TMAO in diabetes pathology remains controversial; while some studies emphasize its detrimental impact on insulin sensitivity and the progression of diabetes-related complications, others suggest potential protective effects. Investigating the largely unexplored role of TMAO’s precursor, trimethylamine, may help elucidate these discrepancies. This review consolidates clinical and experimental findings to clarify TMAO’s complex mechanistic contributions to diabetes pathology.

## Introduction

The management of diabetes and its long-term complications has significantly improved over the past decade with the introduction of new therapeutic interventions aimed at optimizing blood glucose control and weight management, such as sodium-glucose co-transporter-2 inhibitors (SGLT2i) and glucagon-like peptide-1 receptor agonists (GLP-1 RAs), alongside the promotion of healthier lifestyle. This has contributed to improved patient outcomes. However, diabetes remains a significant socioeconomic burden due to its rising prevalence. According to the International Diabetes Federation, nearly half a billion people worldwide are currently living with diabetes, and this figure is projected to increase by 51% by 2045 [1]. Therefore, continuous efforts are being made to fully uncover the underlying pathophysiological processes and to identify new intervention targets or markers for recognizing high-risk individuals.

Recent research on the role of gut microbiota in health and disease has grown rapidly. Studies have demonstrated associations between gut microbial dysbiosis and type 1 diabetes [2], type 2 diabetes [3], and insulin sensitivity [4]. However, the underlying mechanisms and causal relationships remain to be fully established.

Among the potential mediators of microbiota-host interactions, trimethylamine N-oxide (TMAO), a derivative of bacterial metabolism, has garnered significant scientific interest once it has been linked to increased cardiovascular risk [5]. Since then, numerous experimental and clinical studies have explored TMAO’s as a marker and mediator of pathological and/or adaptive processes. Similarly, research on the role of TMAO in the risk assessment of diabetic patients and its involvement in the pathological processes of diabetes and its complications is growing [6]. However, the data are somewhat conflicting, presenting challenges for the development of new diagnostic, prognostic, or treatment strategies in cardiovascular diseases whether using TMAO as a biomarker or a therapeutic target [7].

The purpose of this review is to summarize the available evidence on the role of TMAO in diabetes, encompassing both clinical and experimental studies. Relevant studies evaluating the association between TMAO and diabetes were searched in electronic databases up to June 2024.

## TMAO metaorganismal pathway

TMAO is a common organic compound found in animals, plants and fungi [8]. In humans, most TMAO is produced in the liver through the oxygenation of trimethylamine (TMA) by flavin-containing monooxygenase-3 (FMO3). TMA is a toxic and odorous byproduct of gut bacterial metabolism of choline, L-carnitine, betaine, and phosphatidylcholine, which are abundant in red meat [9, 10]. Due to this connection, the TMA/TMAO pathway has been postulated as a potential link between red meat consumption and increased cardiovascular risk [10]. However, fish and other seafood, which are considered healthy, provide a direct source of TMAO, as marine animals accumulate large amount of this molecule [11]. Nonetheless, broad-spectrum antibiotic treatment has been shown to reduce TMAO concentrations in the blood of both humans and laboratory animals, supporting the idea that gut bacteria are the primary source of TMAO in the bloodstream. Plasma levels of TMAO typically range from 3 μmol/L in healthy individuals to 40 μmol/L in those with kidney failure [5, 10, 12]. Kidneys actively excrete TMAO [13], which may explain why kidney function is a major determinant of plasma TMAO concentration [14]. Relatively high inter- and intra-individual variations in plasma TMAO level are observed in humans [15], which may be attributed not only to kidney function but also to amount of dietary choline, carnitine, TMA, and TMAO, as well as metabolic activity of gut microbiota or FMO3.

Numerous experimental studies have been performed to determine whether TMAO promotes cardiovascular disease. TMAO supplementation or its dietary precursors have been shown to increase atherosclerotic plaque formation in mice [16, 17]. However, some studies contradict these findings, with Collins et al. even suggesting a protective effect of TMAO against atherosclerosis [18]. TMAO has also been shown to increase heart failure severity in mice, with some research indicating that it may induce cardiac fibrosis and disrupt heart energy metabolism [19–21]. Despite this, studies by Querio et al. [22] and others [23] do not support the damaging effects of TMAO on cardiomyocytes. Therefore, it remains unclear whether elevated TMAO levels in cardiovascular disease reflect its harmful nature or if they are merely a confounder. Alternatively, the increase in TMAO could be part of an adaptive response [24].

It is important to note that TMAO is an osmolyte—a small compound capable of maintaining cell volume and counteracting the protein-destabilizing effects of osmotic and hydrostatic stresses. Deep-sea animals utilize this property of TMAO [25], however, human tissues are subjected to high osmotic and hydrostatic pressures as well. For instance, cells in the kidney medulla function in an environment where osmolarity is three to five times higher than that of plasma. To produce concentrated urine, the kidneys accumulate sodium and urea, creating a high osmotic gradient in the medullary region. It has been reported that kidneys also accumulate TMAO, which may act as an osmotic agent and protect kidney cell proteins from disturbances caused by urea [26, 27].

Osmotic stress also plays a role in pathological conditions such as diabetes, where hyperglycemia can cause significant osmotic changes. In fact, diabetes-associated disturbances in osmoregulation are very likely to contribute to the development of cellular dysfunctions and late diabetic complications [28]. Research has demonstrated that diabetes alters the concentration of osmolytes, e.g. decreases taurine and increases sorbitol intracellular accumulation [28]. Arguably, changes in TMAO concentration observed in diabetes may also reflect these shifts in osmolyte levels. While it remains to be determined whether this is an adaptive response, TMAO has been shown to act as a chemical chaperone in diabetes, a topic that will be explored in more detail later.

## Plasma and urine TMAO excretion is increased in diabetes

Although observational studies do not provide direct evidence of causal relationships, identifying biomarkers associated with disease across various patient cohorts is a crucial step in establishing new intervention targets or risk markers. Elevated TMAO levels in diabetes have been reported as early as 1998, when Messana et al. demonstrated higher TMAO concentrations in the urine of diabetic patients, independent of metabolic control, glucosuria, and HbA1c levels [29]. Since then, a number of studies have shown the association between plasma TMAO concentration and diabetes in various cohorts, including population-based [30] as well as different cardiovascular disease cohorts [31, 32]. In 2019, Zhuang et al. conducted a meta-analysis that included 12 studies with a total of 15,314 participants, revealing a dose-dependent association between circulating TMAO levels and diabetes [33]. The analysis included patients with stable coronary artery disease [34, 35], heart failure [36, 37], patients after acute coronary event [38], patients undergoing cardiovascular surgery [39] or coronary angiography [40], as well as population-based [30, 41] and several diabetes case-control studies [42–44]. Among these, only one study reported no difference in TMAO levels between diabetic and non-diabetic patients [35].

Subsequent studies have largely confirmed the meta-analysis findings, though some inconsistencies persist. Namely, TMAO levels were associated with diabetes in coronary heart disease patients [45], in obese population [46], in patients at a high risk of cardiovascular events [47], two large cohorts of general population [48, 49] and smaller case-control study [50]. Additionally, TMAO plasma concentrations were found to be higher in patients with type 2 diabetes compared to both healthy controls and patients with acute myocardial infarction [51]. In fact, despite the fact that TMAO was initially recognized with respect to atherosclerosis, there are studies showing no significant association between TMAO and atherosclerotic cardiovascular disease, and suggesting it is the presence or absence of diabetes that has a decisive effect on plasma TMAO levels [48, 52]. In this regard, Yu et al. directly revealed that a history of diabetes magnified the association between TMAO and coronary heart disease, with high TMAO levels associated with odds ratios of 6.21 and 1.56 in diabetic and non-diabetic subjects, respectively [53].

However, some studies demonstrated that TMAO precursors like choline [54] or TMA [55], rather than TMAO itself, are more significantly associated with type 2 diabetes. Huo et al. showed that the TMAO/TMA ratio decreased gradually during the transition from healthy control through T2DM without microalbuminuria to T2DM with microalbuminuria [55]. In fact, virtually none of other above-mentioned studies measured the concentration of TMA so it may be speculated that TMA would turn out to be a better marker of diabetes than TMAO. Additionally, TMAO may not be a suitable marker for diabetes in certain populations, as demonstrated in HIV patients [56]. Last but not least, the vast majority of studies focus on type 2 diabetes, with much weaker evidence supporting an association between TMAO and other types of diabetes. In fact, to the best of our knowledge, currently there are no studies directly demonstrating TMAO elevation in patients with type 1 diabetes. Although some studies [32] included diabetes patients in general, according to definition of diabetes provided by WHO [57], it should be noted that type 2 diabetes accounts for more than 90% of all diabetes cases [58]. Consequently, the demonstrated associations may not be as significant for type 1 diabetes patients.

Several studies have also examined the correlation between TMAO and gestational diabetes mellitus (GDM). A study by Lin et al. found that TMAO levels in maternal and cord plasma were elevated in women with GDM. Intriguingly, the study suggested a protective role for TMAO in promoting placental and fetal development by inhibiting neutrophil extracellular trap (NET) formation [59]. Another study reported an association between TMAO levels in maternal blood, but not in cord plasma, with GDM [60]. Conversely, Barzilay et al. observed differences in TMAO concentrations between the GDM and control groups only in cord blood, not in maternal blood [61]. On the other hand, two other research groups found no association between TMAO and GDM [62, 63]. Furthermore, Huo et al. demonstrated that it was TMA that was linearly associated with GDM, whereas TMA precursors and TMAO were inversely associated with GDM. Interestingly, the authors suggested that the association between TMAO and the risk of GDM may be *U*-shaped [64].

Observational studies play a crucial role in identifying associations; however, they are subject to significant limitations. One major limitation is the presence of confounding factors, as variables such as dietary patterns, kidney function, age, and comorbidities can significantly influence TMAO levels. Additionally, the issue of reverse causality must be considered, wherein disease states may influence TMAO production or clearance, rather than TMAO being the causal factor in disease development. Furthermore, substantial heterogeneity across studies complicates the interpretation of results. Variability in study populations, methods used to measure TMAO, and definitions of health outcomes contribute to inconsistencies, making it challenging to draw definitive conclusions from observational research.

## TMAO and risk of diabetes

With the growing, albeit inconsistent, evidence linking TMAO with diabetes, research is increasingly focused on evaluating TMAO’s potential as a risk biomarker for incident diabetes (see Table 1). Prospective cohort studies address many limitations of cross-sectional research and show temporal relationships between baseline TMAO levels and subsequent diabetes development. Regarding type 2 diabetes, two research groups identified a positive association between higher baseline TMAO levels and an increased risk of developing diabetes [65, 66]. In the study by Huang et al., both initial TMAO levels and long-term changes in TMAO were associated with incident type 2 diabetes [66]. However, three other studies found no such association [67, 68], although TMAO correlated with markers of insulin sensitivity (HOMA2-IR, fasting insulin). An analysis by Friedrich et al. suggests that sex differences may contribute to these discrepancies. They found that increased TMAO levels were associated with a higher risk of diabetes, but only in women, not in men [69]. Complicating matters further, Papandreou et al. reported a negative association, indicating that higher TMAO levels were linked with a lower risk of future diabetes [70]. This contradictory finding highlights the complexity of TMAO metabolism and suggests potential protective mechanisms that may operate under certain conditions. Also, high intra- and inter-individual variability of TMAO concentrations in diabetic patients may partially explain these inconsistent observations [71].

**Table 1:** Summary of the prospective studies evaluating the association of baseline TMAO with incident diabetes.

Study	Type of diabetes	Study population	Mean follow-up duration	TMAO association with incident diabetes risk	Comments
Svingen et al. [67]	Type 2 diabetes	Nondiabetic individuals with suspected stable angina pectoris	7.5 years	No association	TMAO correlated positively with HOMA2-IR.
Friedrich et al. [69]	Type 2 diabetes	Nondiabetic individuals	5 years	Higher risk in women: ROC^AUC^ = 0.903; No association in men	Sex differences
Papandreou et al. [70]	Type 2 diabetes	Nondiabetic individuals at high risk of cardiovascular disease	3.8 years	Lower risk (HR = 0.52)	
Li et al. [65]	Type 2 diabetes	Nondiabetic individuals in middle-aged and older adults	8.9 years	Higher risk (HR = 1.42)	TMAO was associated with fasting glucose but not with HbA1c, insulin or HOMA-IR.
Lemaitre et al. [68]	Type 2 diabetes	Nondiabetic individuals at high risk of cardiovascular disease	12.1 years	No association	TMAO was associated with fasting insulin.
Huang et al. [66]	Type 2 diabetes	Nondiabetic individuals aged above 35 years	1.85 years	Higher risk (OR = 8.68)	Both initial serum TMAO levels and long-term serum TMAO changes were associated with incident T2D.
Sawicki et al. [78]	Type 2 diabetes	Nondiabetic individuals; two cohorts	3 years and 6 years for different cohorts	No association	TMAO was associated with higher fasting glucose.
Li et al. [72]	GDM	Nondiabetic pregnant women, diagnosed with Gestational Diabetes Mellitus (GDM)	4–12 weeks	Higher risk (OR = 1.22)	

Similarly, studies evaluating the risk of developing GDM in pregnancy reported contradictory results [72, 73]. Moreover, Liu et al. found that it was actually TMA, rather than TMAO, that was exaggerating the risk of GDM [73], once again highlighting this direct TMAO precursor as a potentially more reliable biomarker candidate.

Importantly, the conflicting results surrounding TMAO provide no definitive insight into its causal role in diabetes pathogenesis. To address this, Jia et al. conducted a bidirectional Mendelian randomization analysis to explore the potential causal effect of TMAO on diabetes. Despite the limitations of Mendelian randomization like genetic pleiotropy, it can still offer a more robust estimate of causality than conventional observational studies. Utilizing summary data from genome-wide association studies, they found that genetically predicted higher TMAO levels were not associated with an increased risk of type 2 diabetes or other cardiometabolic diseases. Instead, they observed reverse causality, suggesting that it is diabetes and kidney disease that elevate TMAO levels [74]. Miao et al. proposed the mechanism of this reverse causality showing that insulin resistance upregulates the activity of FMO-3 and hence increases TMAO levels. Specifically, they demonstrated that one of insulin’s most significant effects on the mouse liver in vivo is to suppress FMO3 and TMAO, a function that is impaired in insulin-resistant states [75]. Therefore, TMAO may be an indicator of hepatic insulin resistance, which partially explains the TMAO-associated risk of cardiovascular events and diabetes. Although studies have often adjusted for obesity-related factors, such as body mass index and diabetes, these adjustments likely do not fully encompass the impact of hepatic insulin resistance [76]. The identification of TMAO as a potential biomarker of hepatic insulin resistance offers a novel perspective on its clinical utility, suggesting it may provide information about specific metabolic dysfunctions that complement traditional diabetes markers.

Adding to this already complex picture, it has been repeatedly reported that, unlike TMAO and choline [77], another TMAO precursor, betaine, is negatively correlated with diabetes risk [49, 61, 78]. This raises the interesting possibility that diabetes may alter gut microbiota metabolism of these compounds, potentially inhibiting the two-step oxidation of choline to betaine, consequently enhancing the formation of TMA from choline.

## TMAO and cardiovascular risk in diabetic patients

Diabetes significantly increases the risk of atherosclerotic cardiovascular disease compared to the general population [79]. Given the growing data regarding linkage between TMAO and cardiovascular disease, the role of this metabolite in identifying high-risk individuals within the diabetic population warrants close examination. Substantial evidence point to TMAO as a very promising cardiovascular risk biomarker [80, 81]. However, in the context of diabetes, data has yielded rather mixed results, offering no clear consensus on this matter.

Table 2 summarizes the available studies exploring the relationship between TMAO and cardiovascular risk in individuals with diabetes. Several retrospective observational studies have demonstrated a correlation between elevated TMAO levels and an increased risk of various adverse cardiovascular outcomes in people with type 2 diabetes [38, 82, 83]. These findings have been corroborated by several prospective cohort studies [44, 84, 85]. Interestingly, some research suggests that diabetes may amplify the relationship between elevated TMAO levels and increased cardiovascular risk [38, 86]. Conversely, other studies have reported that this association does not hold true for individuals with a history of diabetes [87, 88]. In line with these latter findings, some studies conducted on diabetic cohorts have found no significant association between TMAO levels and cardiovascular risk [89–92]. For instance, Schrauben et al. demonstrated that neither plasma TMAO levels nor the fractional excretion of TMAO were linked to an increased incidence of atherosclerotic cardiovascular disease or heart failure [90].

**Table 2:** Summary of the studies evaluating the association of TMAO with cardiovascular risk in diabetes.

Study	Population	Outcome	Associations with TMAO	Comments
Tang et al. [44]	Patients with type 2 diabetes, significant proportion of individuals with CVD	3-year MACE 5-year mortality	HR 3.03 HR 3.63	There was no strong correlation between TMAO and glycemic control.
Lever et al. [38]	Patients after acute coronary event with type 2 diabetes	Mortality myocardial infarction heart failure unstable angina all CV events	HR 2.7 HR 4.0 HR 4.6 HR 9.1 HR 2.0	In subjects without diabetes TMAO was only significant for death and heart failure.
Croyal et al. [84]	Patients with type 2 diabetes with no evidence of nondiabetic renal disease	MACE Mortality	HR 1.32 HR 1.75	Plasma TMAO concentrations were not associated with insulin resistance.
Winther et al. [96]	Individuals with type 1 diabetes	Mortality Coronary events CVD events End-stage renal disease (ESRD)	HR 1.19 HR 1.21 HR 1.17 HR 1.67	TMAO was not independently associated with cardiovascular and renal outcomes after adjusting for baseline eGFR.
Flores-Guerrero et al. [82]	Patients with type 2 diabetes	CV mortality	HR 1.93	
Winther et al. [92]	Individuals with type 2 diabetes and albuminuria	Mortality CVD mortality Risk of CVD	no association no association no association	
Sapa et al. [83]	Population of individuals with type 2 diabetes and chronic kidney disease	CV mortality Mortality Incident kidney failure with replacement therapy	HR 1.38 HR 1.13 no association	The lower ratio of urine to plasma TMAO concentration but not baseline plasma TMAO was associated with cardiovascular mortality and all-cause mortality.
Eyileten et al. [86]	Individuals with acute coronary syndrome who underwent percutaneous coronary intervention, with type 2 diabetes	CV morality	HR 11.62	
Bao et al. [87]	Individuals newly diagnosed with coronary heart disease, with 48.1% having diabetes	Severity of CAD (correlation with SYNTAX score) Subgroup with diabetes	β = 0.179 No association	The correlations between TMAO and SYNTAX score did not hold true for subjects who were with histories of diabetes. No adjustment for GFR.
Cardona et al. [89]	Population of individuals with type 2 diabetes and high atherosclerotic cardiovascular disease risk	MACE	no association	TMAO was not significantly associated with cardiovascular outcomes like MACE, CV death, or revascularization, TMAO levels were not influenced by renal function (eGFR).
Schrauben et al. [90]	Individuals with type 2 diabetes and chronic kidney disease	Atherosclerotic CVD events Incident heart failure events	no association no association	
Yu et al. [91]	Individuals with type 2 diabetes who underwent coronary angiography	Risk of triple vessel disease SYNTAX score >22 Presence of CAD	no association no association no association	No association in models adjusted for eGFR; significant correlation with increased coronary atherosclerotic burden was only observed in patients with reduced kidney function (eGFR <60 mL/min/1.73 m²).
Wargny et al. [95]	Individuals with type 2 diabetes	Heart Failure Requiring Hospitalization (HFrH) HFrH and/or CV mortality All-cause mortality	HR 1.32 HR 1.31 HR 1.20	No significant associations in fully adjusted models.
Winther et al. [96]	Individuals with type 1 diabetes	All-cause mortality CV mortality Combined CVD Coronary events End-stage renal disease (ESRD)	HR 1.00 HR 1.00 HR 1.06 HR 1.03 HR 1.41	The associations became insignificant after adjusting for baseline eGFR.

*CAD* coronary artery disease, *CV* cardiovascular, *CVD* cardiovascular disease, *MACE* Major Adverse Cardiovascular Events.

A critical factor to consider is the strong association between plasma TMAO levels and kidney function, which has been well-documented in patients with type 2 diabetes [93, 94]. This association represents a significant confounding factor in TMAO-based risk assessment, as reduced kidney function is independently associated with both elevated TMAO levels and increased cardiovascular risk. Consequently, it is not surprising that many studies observe an attenuation or even a complete loss of the previously observed associations between TMAO and cardiovascular risk after adjusting for estimated glomerular filtration rate (eGFR). For example, in a large prospective cohort study, Wargny et al. initially found that TMAO was associated with an increased risk of heart failure requiring hospitalization. However, after adjusting for eGFR, the association became insignificant [95]. A similar observation was reported by Winther et al. in patients with type 1 diabetes, where the association between TMAO levels and mortality or cardiovascular events was abolished after accounting for baseline eGFR [96].

In summary, while elevated TMAO levels are frequently associated with heightened cardiovascular and renal risks in diabetic patients, the strength and independence of these associations vary depending on the study population and the adjustments made for other risk factors. Especially, stratification approaches that incorporate both TMAO levels and eGFR may provide more accurate risk prediction than either parameter alone.

## TMAO and other diabetic complications

Recent research has also explored the relationship between TMAO and various diabetic complications and comorbidities beyond cardiovascular outcomes. TMAO levels have been found to be significantly higher in patients with diabetic retinopathy compared to those with nondiabetic retinopathy, healthy controls, and those with type 2 diabetes mellitus without retinopathy [97]. Additionally, elevated plasma TMAO levels have been associated not only with an increased risk of developing diabetic retinopathy but also with greater severity of the condition in patients with type 2 diabetes [98]. Although further research is needed to establish causality, a study by Xue et al. suggests that elevated TMAO in patients with proliferative diabetic retinopathy may exacerbate retinal dysfunction, as demonstrated in vitro using retinal cells [99].

Beyond retinopathy, TMAO has also been linked to other diabetic complications. For instance, higher TMAO levels have been associated with increased stroke severity [100] and mild cognitive impairment in the diabetic patients [101]. TMAO levels have also been correlated with osteoporotic complications, including low bone mineral density, osteoporosis, and osteoporotic fractures in patients with type 2 diabetes [102]. However, contrasting findings have been reported, with some studies suggesting that TMAO may protect against bone mineral density reduction during weight loss, independent of dietary interventions and baseline diabetes risk factors [103].

**Fig. 1:**
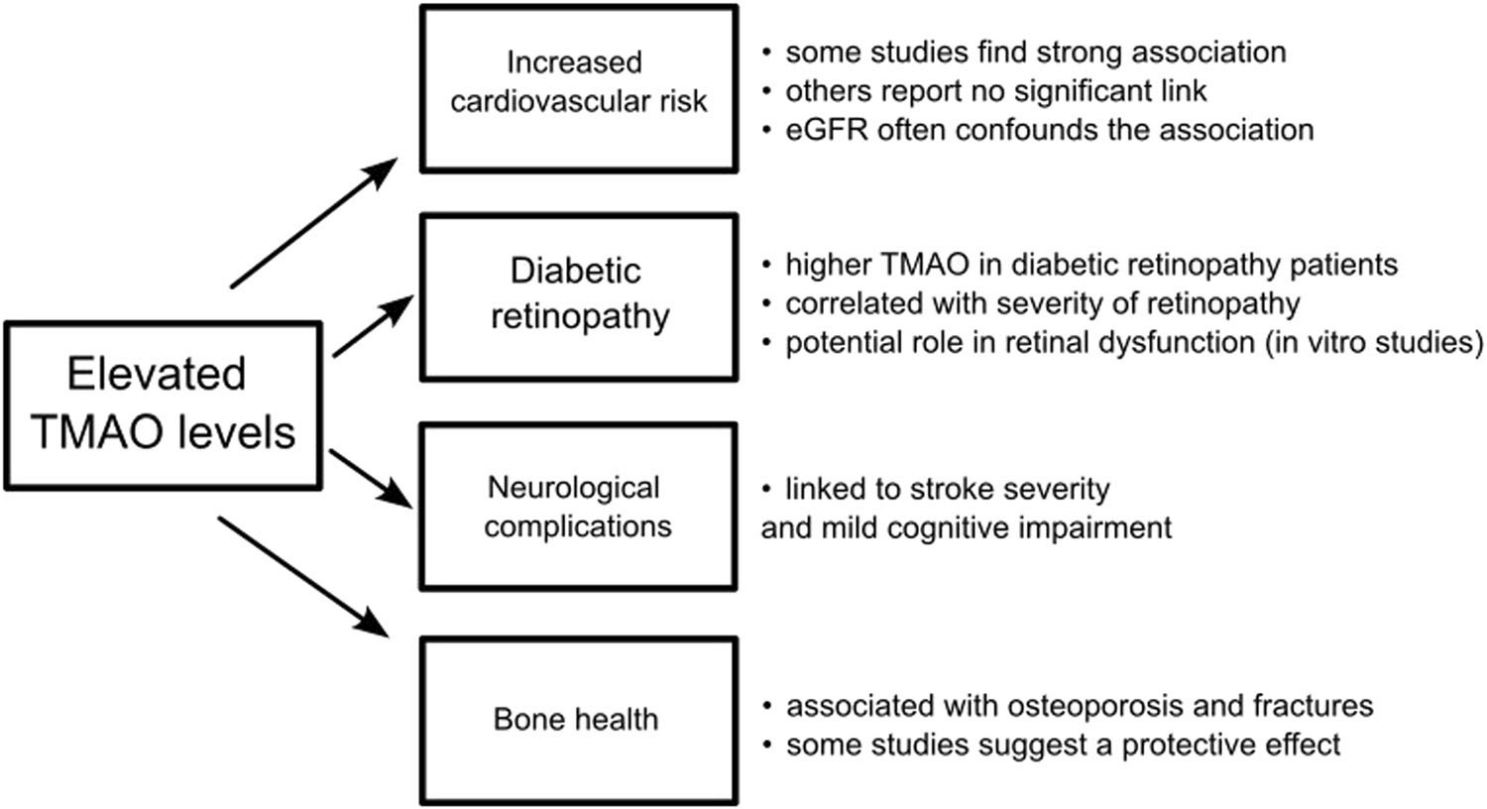
The flowchart summarizing the link between TMAO levels and diabetic complications. *TMAO* trimethylamine N-oxide.

Figure 1 summarizes links between elevated TMAO levels and diabetic complications.

## Impact of pharmacological treatments and lifestyle interventions on plasma TMAO Levels

The concentration of TMAO in the body can be influenced by various therapies and lifestyle changes. In the context of diabetes, special consideration should be given to drugs like metformin and SGLT2 inhibitors, which are key therapeutic agents in the treatment of this condition. Studies indicate that while metformin effectively lowers TMAO levels in mice [104, 105], it may conversely increase these levels in humans [106, 107]. In patients with newly diagnosed type 2 diabetes, treatment with DPP-4 inhibitor does not significantly affect gut microbiota composition, whereas metformin promotes bile acid production, which may enhance the microbiota’s capacity to produce TMAO precursors [106]. On the other hand, CCL4 inhibition leads to changes in gut microbiota and normalizes the lipid profile, ultimately reducing TMAO levels [108]. GLP-1 receptor agonists and SGLT2 inhibitors can also lower TMAO levels while simultaneously improving metabolic control [109, 110]. However, inhibiting SGLT2, when used after myocardial infarction, may paradoxically increase TMAO levels [111].

Lifestyle interventions, which are a key component of diabetes management, also appear to regulate TMAO levels. High-intensity exercise [50], and a vegan diet have been shown to lower TMAO levels, positively impacting cardiovascular health [50, 112, 113]. In contrast, a Western diet, rich in fats and meat, tends to increase this metabolite [106, 114, 115], although fish consumption was associated with significantly greater increases in circulating TMAO than consumption of eggs or beef [116]. Interestingly, some data suggest that Mediterranean diet, along with interventions aimed at increasing the intake of fish, vegetables, and whole grains, does not significantly affect TMAO levels [106, 115, 117, 118]. This suggests that TMAO cannot be regarded as a universal biomarker for cardiometabolic risk independent of dietary factors.

Given the frequent co-occurrence of obesity with type 2 diabetes, the impact of bariatric surgery on TMAO levels also deserves attention. One study indicated that vertical sleeve gastrectomy (VSG) results in reduced TMAO levels [119], while other studies have found that both VSG and Roux-en-Y gastric bypass (RYGB) significantly reduce body weight without necessarily lowering TMAO levels [106, 117]. In some cases, these surgeries even resulted in increased TMAO levels, particularly in patients with type 2 diabetes following RYGB surgery [120]. Other research suggests that TMAO levels may decrease in the long term, depending on the type of surgery and individual patient characteristics [112]. Additionally, there are potentially more effective alternatives to invasive treatments, such as phospholipase A2, group 1B (PLA2G1B) enzyme inactivation and the use of its inhibitors, which have been shown to effectively reduce TMAO levels and offer metabolic health benefits [119].

In conclusion, the varied effects of therapeutic and dietary interventions on TMAO levels underscore the importance of considering diet, lifestyle, and diabetes treatment context when evaluating TMAO’s role as a cardiometabolic risk marker.

## The role of TMAO in diabetes – harmful or protective?

The association between TMAO and diabetes risk raises critical questions about its role in disease etiology. Specifically, it remains unclear whether TMAO actively contributes to the development of diabetes, functions as an adaptive response, or merely reflects underlying pathological processes. This ambiguity has fueled increasing scientific interest in investigating the impact of TMAO on glucose homeostasis.

Several studies suggest that the molecule may play a detrimental role in diabetes. For instance, TMAO treatment has been shown to worsen kidney function, inflammation, and fibrosis in rats with diabetic kidney disease [121].

Additionally, TMAO has been shown to exacerbate diabetic retinopathy by enhancing the proliferation, migration, and tube formation of retinal cells. It also disrupts vascular integrity and impairs tight junctions, further contributing to the progression of retinal vascular complications [122]. Gao et al. further demonstrated that TMAO impairs glucose tolerance, disrupts hepatic insulin signaling pathways, and induces inflammation in adipose tissue in mice fed a high-fat diet [123, 124]. Supporting these findings, a recent study by Kong et al. revealed that TMAO directly decreases glucose-stimulated insulin secretion (GSIS) in MIN6 cells and primary islets from both mice and humans. The study further demonstrated that prolonged exposure to a TMAO precursor, choline, induced significant β-cell dysfunction. This included endoplasmic reticulum (ER) stress, dedifferentiation, apoptosis, and the disruption of β-cell transcriptional identity, highlighting its potential role in impairing β-cell function. Interestingly, reducing TMAO levels by knocking down FMO3 improved β-cell GSIS, increased β-cell proportion, and enhanced glucose tolerance in both male db/db mice and mice fed a choline-enriched diet [125]. Regarding the potential mechanisms of TMAO’s action, it has been suggested that TMAO can directly bind to and activate protein kinase R-like endoplasmic reticulum kinase (PERK), an ER stress kinase, which subsequently induces Forkhead box protein O1 (FoxO1). Inhibition of FMO3, which reduces TMAO levels, has been shown to decrease PERK and FoxO1 activation, ultimately improving glucose tolerance [126]. Similarly, other studies have reported that TMAO induces the PERK-EIF2α-ER stress signaling axis in ex vivo slices and db/db mice [127] and that administration of a Chinese herb ameliorated diabetic cardiomyopathy in mice by reducing serum TMAO levels and suppressing the TMAO/PERK/FoxO1 signaling pathway [128].

Conversely, compelling evidence also suggests a protective role for TMAO in the context of diabetes. Notably, chronic subcutaneous administration of TMAO has been shown to enhance glucose tolerance in mice on a high-fat diet. Additionally, TMAO directly stimulates insulin secretion in isolated pancreatic islets, indicating potential benefits in glucose regulation [129]. While these findings appear to contradict previously mentioned studies, they align with earlier research by Bai et al. from 1998, which demonstrated that subcutaneous or intraperitoneal TMAO injections lower blood glucose levels [130]. These results also support the growing body of evidence that highlights TMAO’s role as a chemical chaperone, capable of reducing endoplasmic reticulum (ER) stress in β-cells. Specifically, TMAO has been found to alleviate ER stress in INS-1 cells [131] and to protect β-cells and primary islet function under diabetic glucolipotoxic conditions by restoring insulin production, insulin granule formation, and insulin secretion [132]. Moreover, TMAO has shown protective effects in liver cells by reducing ER stress and mitigating lipid-induced disruptions in insulin signaling [133]. As a chemical chaperone, TMAO also plays a crucial role in preventing protein misfolding and aggregation, which is particularly relevant in the pathogenesis of diabetes. For instance, TMAO has been demonstrated to counteract the formation of amyloid plaques from human islet amyloid polypeptide (amylin) [134, 135]. Misfolding and aggregation of amylin lead to amyloid fibril formation, causing β-cell dysfunction or death, which contributes to the development of type 2 diabetes [134]. TMAO significantly enhances the formation of properly folded and compact structures of amylin, thereby preventing its harmful aggregation [135]. Evidence also exists that challenges the notion of TMAO exacerbating diabetic complications. For instance, TMAO treatment has been found to mitigate oxidative-nitrative stress in diabetic peripheral neuropathy, suggesting a potential protective effect against certain diabetes-related complications [136] and diabetes-induced impairment in gap junctional communication among astrocytes [137]. Additionally, TMAO has been reported to ameliorate body mass loss and reproductive complications associated with diabetes in rats [138]. Finally, TMAO has been found to improve diabetic wound healing by inhibiting the formation of neutrophil extracellular traps (NETs) and promoting angiogenesis [139], similar to its already mentioned beneficial effects observed in placental and fetal development [59].

What might account for the discrepancies observed in research on the role of TMAO? We propose three potential explanations.

Firstly, in accordance with a fundamental principle of toxicology, the toxicity of a substance is determined by its dose. It is plausible that TMAO functions as an adaptive, protective mechanism up to a certain concentration, beyond which its harmful effects become apparent. For instance, in the aforementioned study by Krueger et al., TMAO at a concentration of 40 µM demonstrated protective effects on islet function. In contrast, β-cells exposed to higher concentrations of 80 and 160 µM exhibited impaired mitochondrial viability and reduced glucose-stimulated insulin secretion (GSIS) [132]. Accordingly, many studies showing its negative effect have used rather supraphysiological doses of TMAO [121–124]. Conversely, Chen et al. suggest that TMAO reduces endoplasmic reticulum stress only at very high (pharmacological) concentrations, such as 300 mM, whereas pathophysiological concentrations (50 mM) do not produce the same effect [126]. All above suggests that TMAO has concentration-dependent biphasic effects.

Secondly, a recent study by Li et al. suggests that the divergent effects of TMAO may be influenced by the route of administration. Specifically, they demonstrated that oral administration of TMAO, but not intraperitoneal injection, resulted in impaired glucose tolerance, elevated lipid levels, and chronic inflammation in adipose tissue [140]. Oral TMAO administration may confound results due to gut microbial conversion to TMA, whereas subcutaneous/intraperitoneal routes isolate TMAO-specific effects. Indeed, virtually all studies showing damaging effect of TMAO have used oral administration of TMAO or its precursors [121–125], while after intraperitoneal or subcutaneous administration only the protective effects were observed [129, 130]. Li et al. propose that after oral gavage, TMAO is converted by gut microbiota into TMA, which is the truly harmful compound. Their findings indicate that TMA, rather than TMAO, may impair glucose tolerance [140]. Arguably, it is a decrease in TMA that is responsible for various positive effects of dimethylbutanol (DMB, an inhibitor of choline to TMA conversion in the gut), such as improved glucose tolerance and insulin sensitivity [141] and decreased the vulnerability of diabetic rats to atrial fibrillation [142], even though many researchers attribute these effects to reduced TMAO levels. Although TMA is increasingly recognized as a damaging agent [23, 143–145], its role in cardiometabolic pathology remains largely unexplored.

Lastly, it has been suggested that FMO3, the enzyme responsible for converting TMA into TMAO, might exert harmful effects independently of TMAO levels [76], as it may directly boost hepatic lipogenesis and gluconeogenesis [146], and support the expression of FoxO1, a key node responsible for the insulin-resistant state [75]. However, other studies indicate the protective effect of FMO3, such as reducing lipid-induced endoplasmic reticulum stress in the liver, independent of insulin signaling pathways [147, 148].

In summary, while some studies suggest that TMAO exacerbates insulin resistance and accelerates disease progression, others propose that it may have protective effects. This discrepancy leaves a key question unresolved: Is TMAO itself pathogenic, or does it simply serve as a biomarker reflecting underlying metabolic dysfunction? It is plausible that elevated TMAO levels primarily indicate hepatic insulin resistance, with their concentrations largely influenced by renal function and dietary intake, rather than playing a direct causal role in the pathogenesis of diabetes.

**Fig. 2:**
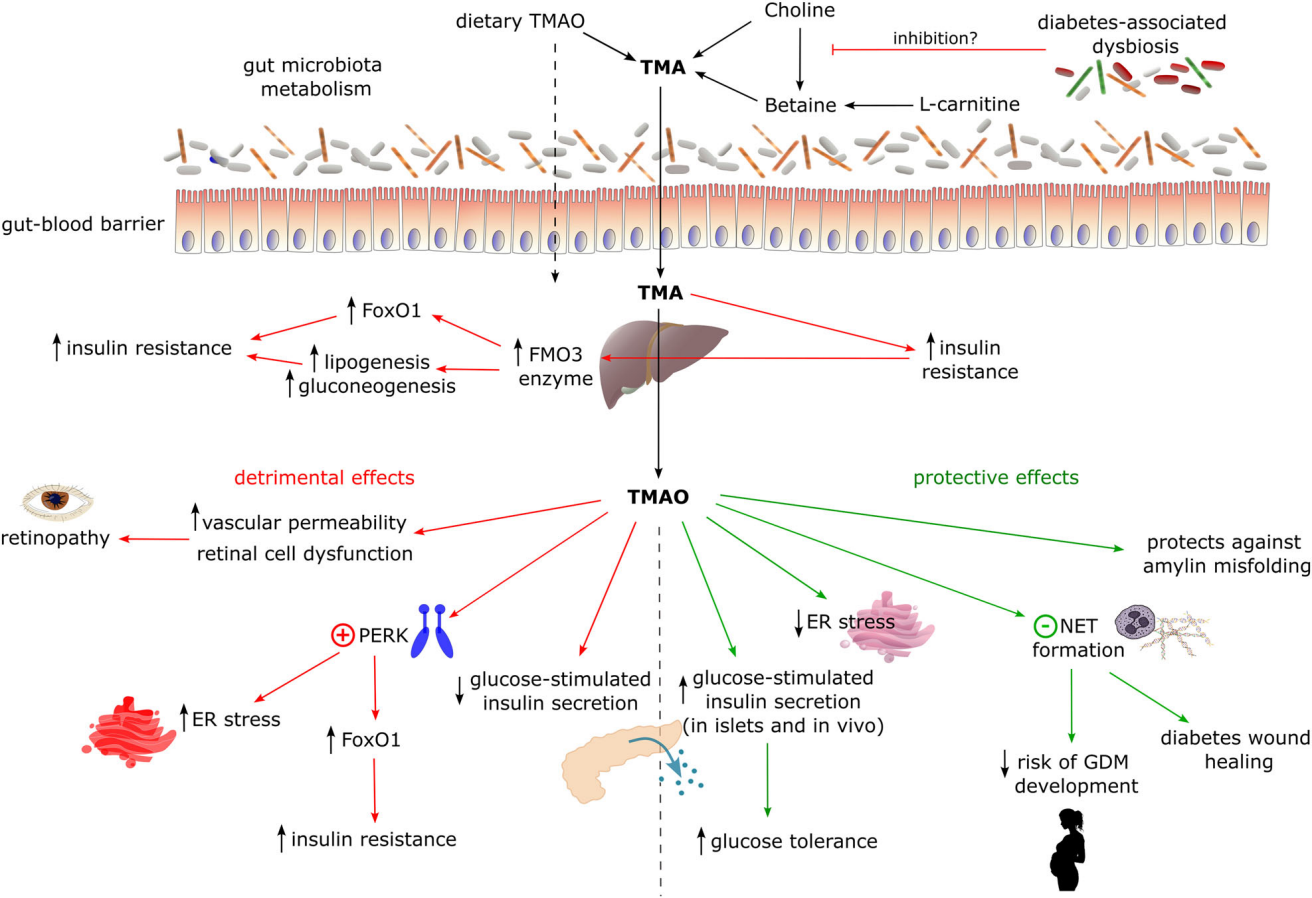
The possible effects of TMA/TMAO/FMO3 on diabetes pathophysiology. Details in the text. ER stress endoplasmic reticulum stress, FoxO1 Forkhead box protein O1, FMO3 Flavin-containing monooxygenase 3, GDM gestational diabetes mellitus, NET neutrophil extracellular traps, PERK protein kinase R-like endoplasmic reticulum kinase, TMA trimethylamine, TMAO trimethylamine N-oxide.

The possible mechanisms regarding TMAO role in diabetes are summarized in Fig. 2.

## Clinical applications

Despite extensive research, the immediate clinical utility of TMAO in diabetes management remains limited due to several factors. The substantial variability in TMAO levels—affected by diet, kidney function, and microbial metabolism—complicates the establishment of standardized reference ranges. Additionally, the bidirectional relationship between TMAO and metabolic dysfunction suggests that elevated levels may reflect a consequence, rather than a cause, of diabetes, reducing its potential as an intervention target.

However, emerging evidence highlights potential applications in specific clinical contexts. In patients with established diabetes, TMAO may serve as a complementary biomarker for hepatic insulin resistance, offering insights not captured by traditional glycemic measures. Furthermore, its association with diabetic complications suggests it could identify high-risk individuals for intensified monitoring and intervention, such as those with elevated TMAO and impaired kidney function who may be at increased risk for cardiovascular complications.

The impact of therapeutic interventions on TMAO metabolism also warrants attention. Research indicates that common diabetes medications—such as metformin, GLP-1 receptor agonists, and SGLT2 inhibitors—affect TMAO levels differently. Understanding these drug-specific effects may help optimize therapeutic strategies.

Several promising research directions may enhance TMAO’s clinical utility. Comprehensive metabolic profiling, simultaneously measuring TMAO, TMA, and related precursors (choline, carnitine, betaine), could improve risk stratification compared to isolated TMAO assessment. The varying associations of these metabolites with diabetes risk suggest that a metabolic signature approach may outperform single biomarker strategies.

Longitudinal studies assessing changes in TMAO levels could clarify whether these fluctuations predict disease progression or therapeutic response, offering more insight than static measurements. Integration with emerging biomarkers of insulin resistance and β-cell function may further refine predictive accuracy.

Finally, the role of TMAO in different diabetes phenotypes warrants further exploration. While most research has focused on type 2 diabetes, data on type 1 and monogenic forms are limited. Understanding whether TMAO associations vary by diabetes subtype could improve risk stratification and uncover distinct pathophysiological mechanisms. Additionally, investigating genetic variants in TMA-TMAO pathway enzymes may identify high-risk subpopulations for targeted intervention.

### Perspectives

Given that patients with diabetes often have elevated levels of TMAO in their blood, the scientific community has been driven by a “gut feeling,” prompting numerous studies to explore the association between TMAO and diabetes risk, as well as its direct effects on diabetes pathophysiology and complications. However, the results have been somewhat conflicting. It is plausible that TMAO is merely an indicator of hepatic insulin resistance, largely influenced by kidney function and diet. The role of TMAO in diabetes pathology remains controversial. Some studies suggest it worsens insulin tolerance and diabetes complications, while others indicate potential protective effects. At the core of the issue lies a fundamental question: What is the true harmful agent—TMAO, its precursor TMA, or the liver enzyme FMO3 itself? This critical question emphasizes the need for further research, particularly regarding the direct effects of TMA on glucose homeostasis.

Currently, there is insufficient evidence to classify TMAO as a direct pathogenic factor in diabetes. However, TMAO shows strong potential as a complementary biomarker for clinical risk stratification, as it may reflect aspects of metabolic dysfunction that are not captured by traditional markers like blood glucose levels.

As research into the role of TMAO in diabetes advances, it underscores the complex relationship between gut microbiota and host metabolism. With improved analytical methods and broader access to metabolic profiling, TMAO and related metabolites could become valuable tools for more personalized approaches to assessing diabetes risk, subtyping, and monitoring treatment responses.
